# Application of the long axial field-of-view PET/CT with low-dose [^18^F]FDG in melanoma

**DOI:** 10.1007/s00259-022-06070-7

**Published:** 2022-12-07

**Authors:** Christos Sachpekidis, Leyun Pan, Annette Kopp-Schneider, Vivienn Weru, Jessica C. Hassel, Antonia Dimitrakopoulou-Strauss

**Affiliations:** 1 Clinical Cooperation Unit Nuclear Medicine, German Cancer Research Center, Im Neuenheimer Feld 280, 69210 Heidelberg, Germany; 2 Department of Biostatistics, German Cancer Research Center (DKFZ), Heidelberg, Germany; 3 Department of Dermatology, Skin Cancer Center, University Hospital Heidelberg, Heidelberg, Germany; 4 National Center for Tumor Diseases Heidelberg, Heidelberg, Germany

**Keywords:** Melanoma, LAFOV PET/CT, [^18^F]FDG, Total body, Whole-body, SUV

## Abstract

**Aim:**

The recent introduction of long axial field-of-view (LAFOV) PET/CT scanners has yielded very promising results regarding image quality and sensitivity in oncological patients. We, herein, aim to determine an appropriate acquisition time range for the new long axial field of view Biograph Vision Quadra PET/CT (Siemens Healthcare) using low dose [^18^F]FDG activity in a group of melanoma patients.

**Methodology:**

Forty-nine melanoma patients were enrolled in the study. All patients underwent total body PET/CT from the top of the head through the feet in two bed positions (field-of-view 106 cm) after i.v. injection of 2.0 MBq/kg [^18^F]FDG. The PET images of the first bed position (head to upper thigh; PET-10) were reconstructed and further split into 8-min (PET-8), 6-min (PET-6), 5-min (PET-5), 4-min (PET-4), and 2-min (PET-2) duration groups. Comparisons were performed between the different reconstructed scan times with regard to the visual evaluation of the PET/CT scans using the PET-10 images as reference and by calculating the 95%-CI for the differences between different time acquisitions. Moreover, objective evaluation of PET/CT image quality was performed based on SUV calculations of tumor lesions and background, leading to calculation of liver signal-to-noise ratio (SNR), and tumor-to-background ratio (TBR).

**Results:**

A total of 60 scans were evaluated. Concerning visual analysis, 49/60 (81.7%) PET-10 scans were pathological, while the respective frequencies were 49/60 (81.7%) for PET-8 (95%-CI: − 0.0602–0.0602), 49/60 (81.7%) for PET-6 (95%-CI: − 0.0602–0.0602), 48/60 (80%) for PET-5 (95%-CI: − 0.0445–0.0886), 46/60 (76.7%) for PET-4 (95%-CI: − 0.0132–0.1370), and 45/60 (75%) for PET-2 (95%-CI: 0.0025–0.1593). In 18 PET-10 scans, the extent of metastatic involvement was very large, rendering the accurate calculation of [^18^F]FDG-avid tumor lesions very complicated. In the remaining 42 PET-10 scans, for which the exact calculation of tumor lesions was feasible, a total of 119 tumor lesions were counted, and the respective lesion detection rates for shorter acquisitions were as follows: 97.5% (116/119) for PET-8 (95%-CI: 0–1), 95.0% (113/119) for PET-6 (95%-CI: 0–1), 89.9% (107/119) for PET-5 (95%-CI: 0–2), 83.2% (99/119) for PET-4 (95%-CI: 1–2), and 73.9% (88/119) for PET-2 (95%-CI: 2–4). With regard to objective image quality evaluations, as a general trend, the reduction of acquisition time was associated with a decrease of liver SNR and a decrease of TBR, although in lesion-based analysis the change in TBR and tumor SUV_mean_ values was non-significant up to 6 and 5 min acquisitions, respectively.

**Conclusions:**

In melanoma, low-dose LAFOV PET/CT imaging is feasible and can reduce the total scan time from head to upper thigh up to 5 min providing comparable diagnostic data to standard lengths of acquisition. This may have significant implications for the diagnostic work-up of patients with melanoma, given the need for true whole-body imaging in this type of cancer.

**Supplementary Information:**

The online version contains supplementary material available at 10.1007/s00259-022-06070-7.

## Introduction

Positron emission tomography (PET) integrated with computed tomography (CT) is a whole-body imaging technique combining functional and morphological information in one procedure. PET/CT, mainly with the radiotracer 2-deoxy-2-[fluorine-18] fluoro-D-glucose ([^18^F]FDG), the workhorse of molecular imaging, is nowadays a standard imaging method applied in the clinical routine of oncology for several indications, including diagnosis, staging, response evaluation, targeting of radiation therapy, disease control and recurrence detection [1, 2]. Particularly, in malignant melanoma, [^18^F]FDG PET/CT is considered the modality of choice when there is clinical suspicion of locoregional or systemic metastases at initial staging, disease recurrence, and for treatment response assessment [3–7].

In most types of tumors, the imaging field of view (FOV) for PET/CT studies is from the skull base to upper thigh (torso imaging), which covers most of the relevant portions of the body in many oncological diseases [1]. However, due to the propensity of malignant melanoma to metastasize anywhere in the body, true whole-body (total body) imaging, i.e., from the top of the head through the feet, is recommended as the standard of care for the assessment of the malignancy [8]. This approach, however, leads to a non-negligible extension of the scanning time with a subsequent decrease in throughput, which is important for busy nuclear medicine departments.

Significant developments have occurred in the field of molecular imaging since the original description of the prototype, fused PET/CT scanner by Beyer et al. [9]. Most recently, long axial field of view (LAFOV) PET/CT systems, such as the Biograph Vision Quadra (Siemens Healthineers) and the uEXPLORER (United Imaging), have become available, rendering digital total-body PET/CT as the current state of the art, enabling, apart from the achievement of larger anatomical coverage, an increase in system sensitivity [10–12]. Indeed, preliminary results from the clinical application of these systems show that they lead to improved image quality and lesion quantification resulting from higher sensitivity, alternatively allowing for a significant reduction in acquisition time or low-dose examination protocols [13–15].

In the present study, we investigated melanoma patients with the new LAFOV Biograph Vision Quadra PET/CT after application of low-dose [^18^F]FDG. Our aim was to determine an appropriate acquisition time range for low-dose oncological imaging by analyzing different emulated scan times and the quality of the resultant PET images.

## Materials and methods

### Patients

A total of 49 patients (31 male, 18 female; mean age 64.9 years) with malignant melanoma were enrolled in this retrospective analysis. All patients underwent [^18^F]FDG PET/CT for staging or re-staging purposes. Seven patients underwent two PET/CT scans, while two of them underwent in total three scans. Thus, in total, 60 PET/CT scans were performed and analyzed. Patients gave written informed consent to participate in the study and to have their medical records released. The study was approved by the Ethical Committee of the University of Heidelberg (S-107/2012) and the Federal Agency for Radiation Protection (Bundesamt für Strahlenschutz, Z 5-22,463/2-2012-016). The patients’ characteristics are summarized in Table 1.

**Table 1:** Baseline patient characteristics

Patient characteristics	*N*
Number of patients	49
Total number of PET/CT scans	60
Median applied dose, MBq	166 (93–263)
Median age, years	64 (35–87)
Gender Male Female	
	31 (63%)
	18 (37%)
Type of primary melanoma	
cutaneous	40 (82%)
mucosal	1 (2%)
uveal	5 (10%)
not defined	3 (6%)
Previous systemic therapy	
yes	26 (53%)
no	23 (47%)
Tumor stage at the time of PET/CT	
I	4 (8%)
II	4 (8%)
III	12 (24%)
IV	26 (53%)
not defined	3 (6%)

### PET/CT examination

All patients fasted for at least 6 h before [^18^F]FDG administration. Patients underwent total body PET/CT (Biograph Vision Quadra, Siemens Co., Erlangen, Germany) after intravenous administration of body weight-adapted dose of 2.0 MBq/kg [^18^F]FDG 60 min post-injection (p.i.). Total-body imaging from the skull through the feet was performed in two bed positions (each FOV 106 cm): the first bed position covered the area from the top of the head to the upper thigh (10-min acquisition in list mode; PET-10), and the second covered the lower extremities (5-min acquisition in list mode). The PET images of the first bed position (head to upper thigh) were first reconstructed using the entire 10-min data and were further split into 8-min (PET-8), 6-min (PET-6), 5-min (PET-5), 4-min (PET-4), and 2-min (PET-2) duration groups to compare different acquisition times for fast acquisition scenarios.

A low-dose attenuation CT (120 kV, 30 mA) was used for attenuation correction of the PET data and for image fusion. All PET images were attenuation-corrected and an image matrix of 440 × 440 pixels was used for iterative image reconstruction. Images were reconstructed using the manufacturer’s (Siemens Healthineers) default reconstruction method, employing the point spread function + time-of-flight algorithm (PSF + TOF, 4 iterations × 5 subsets) without Gaussian filtering into 1.65 × 1.65 × 1.65 mm^3^ voxels.

### Data analysis

#### Visual assessment of PET/CT scans

PET/CT images were evaluated independently on an Aycan workstation by two experienced nuclear medicine physicians well versed in melanoma diagnosis (CS, ADS). In cases of discrepancy, an agreement was reached after mututal consultation. In order to reduce bias, with regard to the same examination, the reading of PET/CT scans for the different time acquisition protocols was performed with an interval of at least 1 week.

Visual analysis was based on the identification of sites of focal, non-physiologic [^18^F]FDG uptake above surrounding background activity, which were considered consistent with melanoma lesions. On the basis of these findings, each scan was classified as normal (no melanoma lesions) or pathologic (at least one [^18^F]FDG-avid melanoma lesion). Moreover, the number of melanoma lesions was determined in each scan, with a maximum of up to 10 measured lesions per patient. With regard to lesion detectability, the results of the 10-min PET acquisition (PET-10) served as the reference, with which the results of the other duration groups (PET-8, PET-6, PET-5, PET-4, PET-2) were compared.

#### Objective evaluation of PET/CT image quality

Objective evaluation of PET/CT image quality was based on volumes of interest (VOIs) and on subsequent calculation of SUV values (SUV_mean_, SUV_max_). Melanoma lesions’ quantification was based on VOIs drawn with an isocontour mode (pseudo-snake) that were placed over tumor lesions using a dedicated software (PMOD Technologies, Zurich, Switzerland) [16]. With regard to melanoma lesions, these were assessed on a patient-basis (‘patient-based analysis’), calculated from all evaluated [^18^F]FDG-avid lesions in each PET/CT scan and averaged in each patient, and on an independent-basis (lesion-based analysis), in which each tumor lesion was evaluated separately.

Due to its reasonably uniform tracer uptake, the liver parenchyma was used for background measurements by the positioning of spheric VOIs in the right liver lobe, if without lesions, and at least 1 cm away from the edge of the liver [1, 17]. Moreover, semi-quantitative evaluations of the blood pool were performed after placement of VOIs in the lumen of the descending aorta in the mediastinum without inclusion of the aortic wall. VOIs were copied and pasted between different images obtained from different (list mode) frame durations, ensuring that the same VOI was analysed for each acquisition as previously described [14]. The signal-to-noise ratio (SNR) of the background (liver) was measured as the SUV_mean_ of the background divided by its standard deviation (SD). Tumour-to-background ratios (TBRs) were defined as SUV_mean_ of the melanoma lesion divided by SUV_mean_ for the liver background [14, 17].

It has to be noted that the comparison of the results, both of visual analysis and objective evaluation of PET/CT image quality, was focused on the first bed position covering the area from the top of the head to the upper thigh.

#### Statistical analysis

Continuous variables were expressed as the mean ± standard deviation (SD). Regarding visual (qualitative) assessment of PET/CT scans, score-based 95%-confidence intervals (95%-CI) for the differences of the proportion of pathological scans between the various PET acquisition times and the reference of PET-10 were calculated using the method of Tango and Tang as implemented in the function pairbinci in the R package ratesci [18–20]. Respectively, concerning the lesion detection rate, the 95%-CI for the 90%-quantile of the differences in number of tumor lesions between the various PET acquisition times and the reference of PET-10 were calculated using the function quantileCI from the R package MKinfer. Further, differences between parameters employed for objective evaluation of PET/CT image quality in the liver and mediastinum were assessed using the paired Student’s *t*-test. For the tumor lesions, in terms of lesion-based analysis the differences were assessed using a linear mixed model to take into account that some lesions belonged to the same patient, with log transformation employed on the quantitative parameters to eliminate skewness observed in the measurements. Respectively, for patient-based analysis, differences were assessed using paired t-test after averaging the lesions measurements for each patient. The different PET/CT scans of some patients examined in different time points (*n* = 11 scans of seven patients) were considered independent in this analysis, since in the meantime the patients had received systemic therapies, which markedly altered the [^18^F]FDG uptake. Statistical significance was considered for *p*-values less than 0.05. Statistical analysis was performed in R (version 4.0.3).

## Results

### Visual assessment of PET/CT scans

Based on the results of PET-10, a total of 49/60 pathological (81.7%) and 11 normal (18.3%) scans were diagnosed. The respective numbers of pathological PET/CT scans were 49/60 (81.7%) for PET-8, 49/60 (81.7%) for PET-6, 48/60 (80%) for PET-5, 46/60 (76.7%) for PET-4, and 45/60 (75%) for PET-2. The 95%-CI for the differences of proportion of the pathological scans among the different, shorter PET acquisitions and the reference of PET-10 were the following: − 0.0602–0.0602 (PET-8), − 0.0602–0.0602 (PET-6), − 0.0445–0.0886 (PET-5), − 0.0132–0.1370 (PET-4), and 0.0025–0.1593 (PET-2).

In 18 PET-10 scans, the extent of hypermetabolic metastatic involvement was very large (> 10 lesions), rendering the exact calculation of [^18^F]FDG-avid tumor lesions very difficult. In these 18 cases, a very large number of lesions (> 10) was also detected with all rest reconstruction protocols, with the exception of one case, in which PET-2 demonstrated less than ten, in paticular eight, tumor lesions. In the remaining 42 PET-10 scans, for which the exact calculation of tumor lesions was feasible, a total of 119 lesions was detected. Compared to the reference of PET-10 images, PET-8, PET-6, PET-5, PET-4, and PET-2 images had lesion detection rates of 97.5% (116/119), 95.0% (113/119), 89.9% (107/119), 83.2% (99/119), and 73.9% (88/119), respectively. The 95%-CI for the 90%-quantile of the differences in number of tumor lesions between the various PET acquisition times and the reference of PET-10 were as follows: 0–1 (PET-8), 0–1 (PET-6), 0–2 (PET-5), 1–2 (PET-4), and 2–4 (PET-2). Notably, the observed differences between PET-10, PET-8, and PET-6 regarding the number of detected lesions, would have no clinical or therapeutic consequences for any of the studied patients, since they would not potentially lead to differences in terms of staging or restaging the disease.

**Fig. 1:**
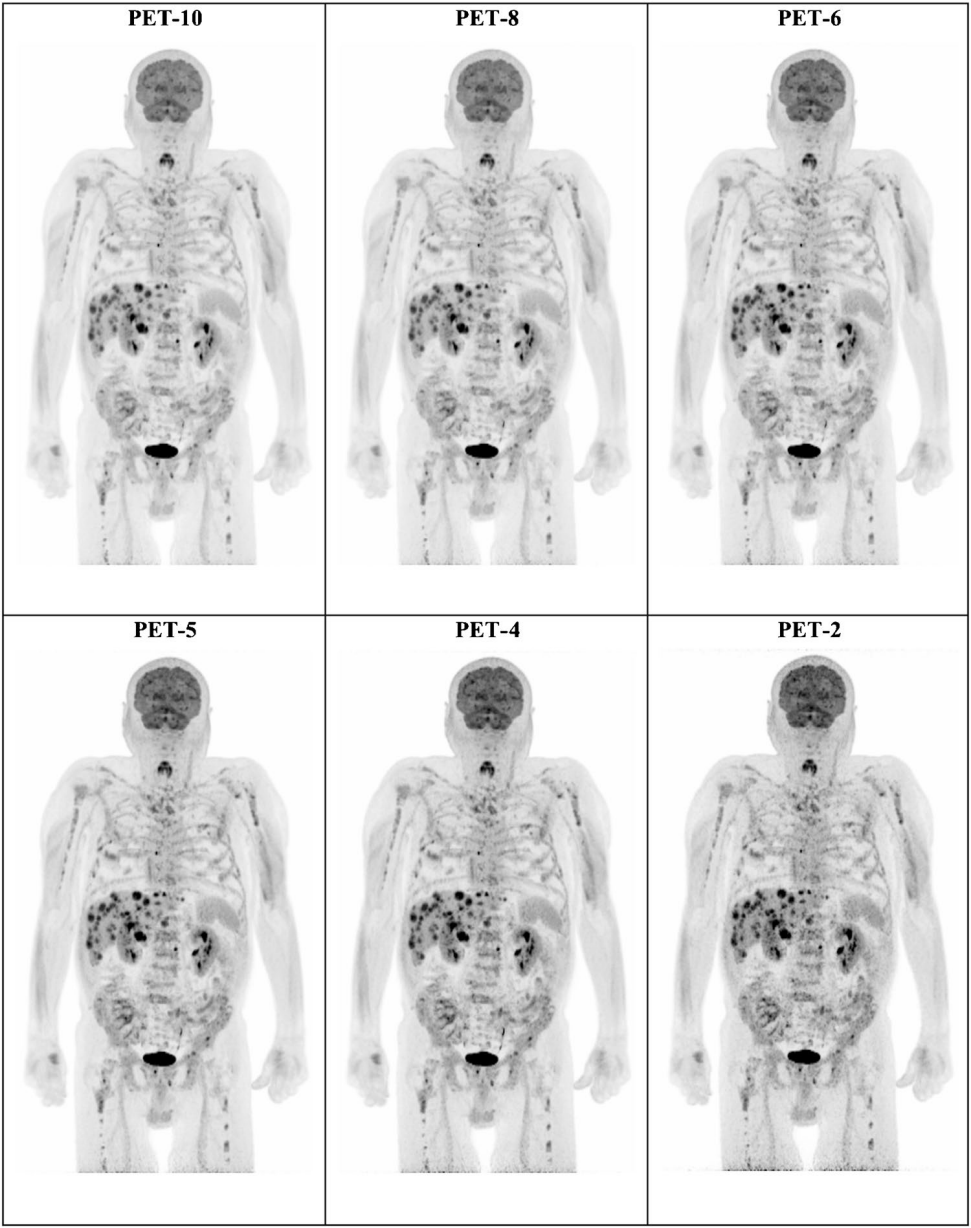
Example of maximum intensity projection (MIP) [^18^F]FDG PET images of a 74-year-old man with stage IV cutaneous melanoma planned for treatment with immune checkpoint inhibitors. Presented are the PET-10, PET-8, PET-6, PET-5, PET-4, and PET-2 acquisitions. All acquisitions clearly demonstrate disseminated [^18^F]FDG-avid metastastic disease

**Fig. 2:**
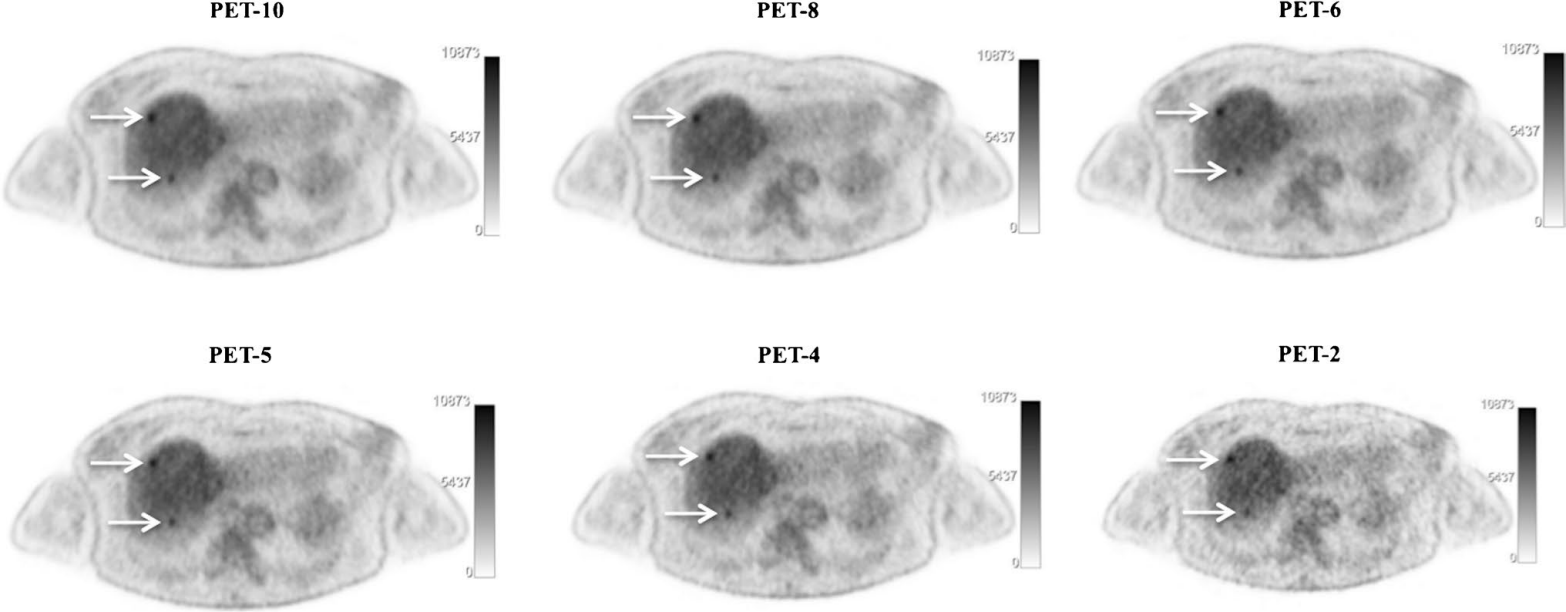
Example of axial [^18^F]FDG PET images of a 61-year-old woman with stage IV uveal melanoma under treatment with tebentafusp. Presented are the PET-10, PET-8, PET-6, PET-5, PET-4, and PET-2 acquisitions. Two focal hypermetabolic, metastatic lesions in the upper part of the liver parenchyma (arrows) clearly depicted in the PET-10, PET-8, PET-6, PET-5, and PET-4 acquisitions. The lesions are, on the other hand, not very distinct in the PET-2 acquisitions, especially the dorsally localized metastasis

The results of the visual assessment of PET/CT scans are presented in Tables 2 and 3. Figures 1 and 2 present examples of [^18^F]FDG PET images of patients assessed with different time acquisition protocols.

**Table 2:** Results of visual analysis of PET/CT images with regard to classification of scans as normal (no melanoma lesions) or pathologic (at least one [^18^F]FDG-avid melanoma lesion)

Measurement	PET-10	PET-8	PET-6	PET-5	PET-4	PET-2
Number of pathological scans	49/60 (81.7%)	49/60 (81.7%)	49/60 (81.7%)	48/60 (80.0%)	46/60 (76.7%)	45/60 (75.0%)
95%-CI for difference of proportions of pathological scans^#^	n.a	-0.0602—0.0602	-0.0602—0.0602	-0.0445—0.0886	-0.0132—0.1370	0.0025—0.1593

^#^Compared to the reference of PET-10

*n.a*. not applicable

**Table 3:** Results of visual analysis of PET/CT images with regard to lesion detection rate

Measurement	PET-10	PET-8	PET-6	PET-5	PET-4	PET-2
Lesion detection rate ^#^	n.a	116/119 (97.5%)	113/119 (95.0)%	107/119 (89.9%)	99/119 (83.2%)	88/119 (73.9%)
95%-CI for 90%-quantile of differences of number of lesions ^#^	n.a	0–1	0–1	0–2	1–2	2–4

After exclusion of the 18 scans in which the exact calculation of tumor lesions was very difficult due to extent of metastatic involvement (> 10 lesions)

*n.a*. not applicable

^#^Compared to the reference of PET-10

### Objective evaluation of PET/CT image quality

**Fig. 3:**
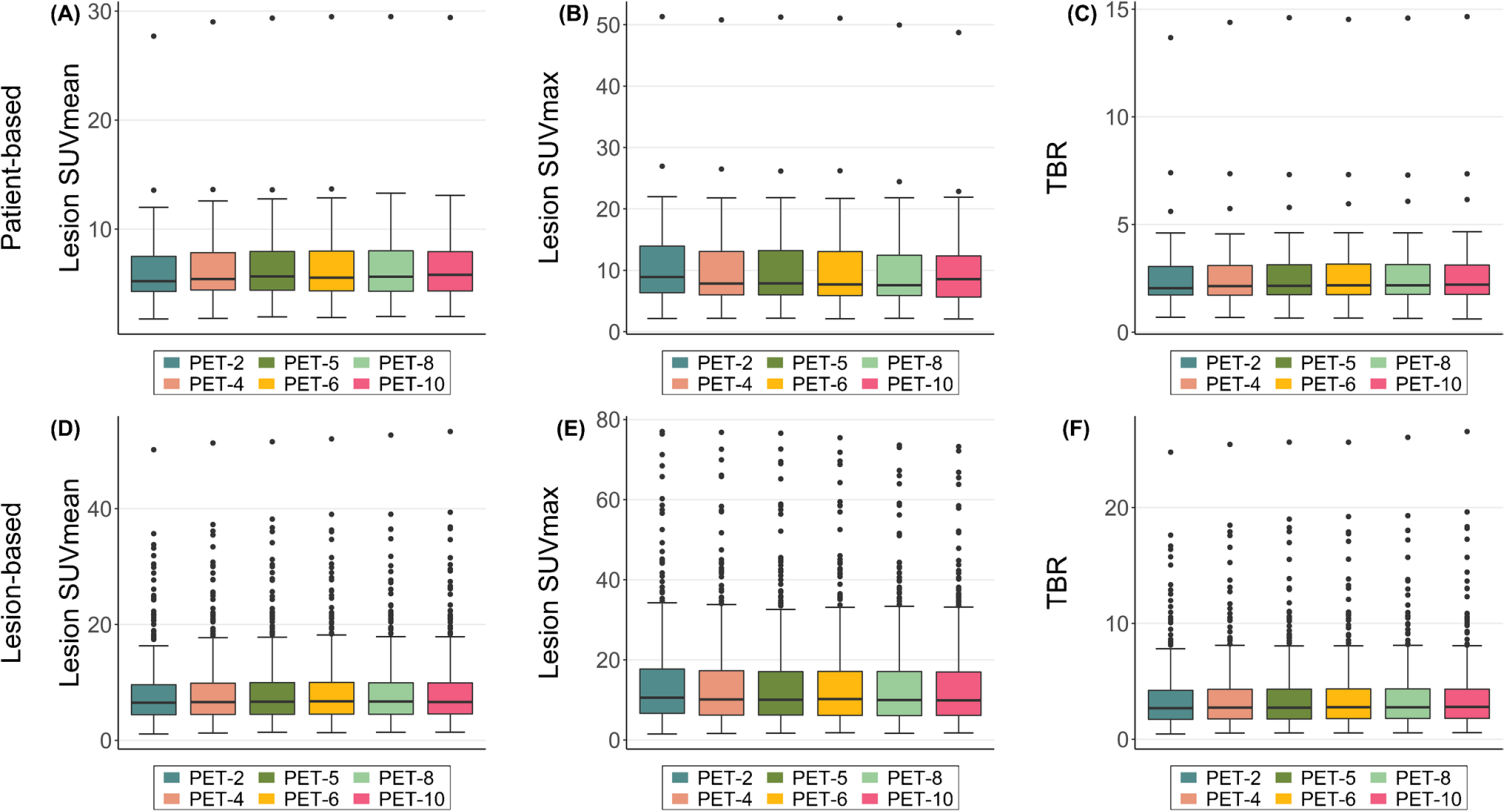
Box plots for comparison of objective image quality parameters in tumor lesions among the different PET acquisition groups in terms of patient-based (**A**–**C**) and lesion-based (**D**–**F**) analysis. As a general trend, SUV_mean_ decreased while moving from longer to shorter-duration protocols, although the changes with the reference 10-min values were non-significant up to 5-min acquisitions (**A**, **C**). SUV_max_ was significantly lower in PET-10 than all other shorter-duration protocols (**B**, **D**). TBR decreased in shorter protocols, although the decrease was non-significant up to 8-min and 6-min acquisitions in patient- and lesion-based analysis respectively (**C**, **F**)

**Fig. 4:**
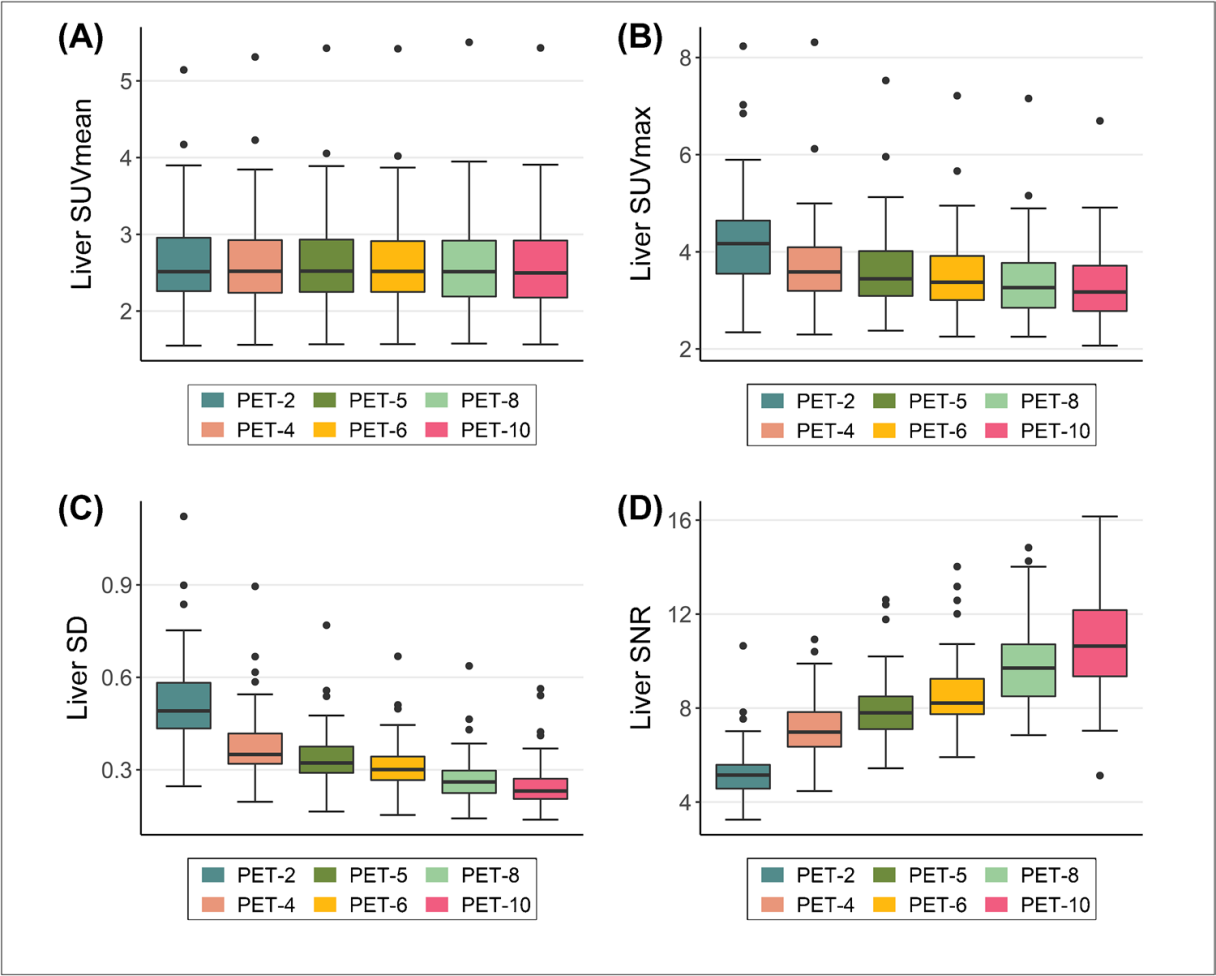
Box plots for comparison of objective image quality parameters in the liver. As a general trend, the liver uptake, SD and image noise progressively increased (**A**–**C**) while the liver SNR decreased (**D**) as acquisition times decreased

**Fig. 5:**
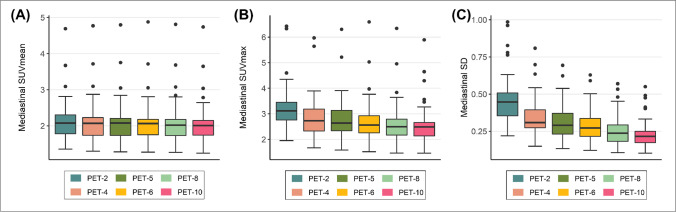
Box plots for comparison of objective image quality parameters in the mediastinum (descending aorta). As a general trend, the blood pool uptake and SD progressively increased as acquisition times decreased (**A**–**C**)

The results of the objective image quality assessments are presented in Figs. 3, 4, and 5 as well as in Supplementary File 1. As a general trend, as the acquisition time decreased the background (liver) uptake and image noise increased, while the liver SNR decreased. In particular, the liver SNR calculated in the PET-10 images was significantly higher than all other acquisitions. Similarly, tracer uptake in the mediastinum progressively increased as PET acquisition duration decreased.

A total of 463 tumor lesions were evaluated quantitavely. Both patient-based and lesion-based analysis revealed for SUV_mean_ no significant differences between 10-min, 8-min, 6-min, and 5-min acquisitions, while in shorter-duration protocols (PET-2, PET-4) SUV_mean_ decreased significantly. Respectively, SUV_max_ was significantly lower in PET-10 than all shorter-duration protocols in both patient-based and lesion-based analysis. Finally, the shortening of acquisition time up to 6 min and 8 min led to no differences regarding TBR in lesion- and patient-based analysis respectively, while in shorter-duration protocols TBR significantly decreased compared to the 10-min protocol.

## Discussion

In standard clinical practice, the achievement of high quality PET images that can overcome the inherent poor sensitivity of current whole-body PET scans requires either an increase in the radiotracer injection dose or a prolongation of the acquisition time, both methods with radiation exposure- and logistical consequences [15, 21]. In this context, the recent advent and clinical introduction of ultra-extended FOV PET/CT scanners represents an evolution in molecular imaging with potentially major clinical and financial implications, while enabling, at the same time, a reduction of the associated radiation burden [22]. Importantly, it has already been shown that low-dose or even ultra-low-dose radiotracer activities achieve a comparable image quality to conventional PET, and meet clinical requirements in different tumors [13–15, 23, 24]. Based on this experience, we, herein, aimed to determine an appropriate acquisition time range for low-dose oncological PET/CT imaging with the new LAFOV Biograph Vision Quadra PET/CT (Siemens Healthcare) after application of 2.0 MBq/kg, and not 3.0 MBq/kg as per EANM guidelines [1], [^18^F]FDG activity in a cohort of melanoma patients.

The major findings of our analysis are the following: firstly, the reduction of PET acquisition times up to 5 min in low-dose [^18^F]FDG LAFOV PET/CT imaging is feasible in melanoma and can be safely performed in terms of visual analysis, applied both in a binary fashion (normal-pathological) and based on calculation of lesion detection rate. In particular, the shortening of PET acquisition times to 6 min was associated with absolutely no potential clinical consequences in the context of patient staging or restaging in the present cohort for any of the studied patients. Further, a decrease of both SNR and TBR is observed by reducing the PET acquisition time, although the change in absolute tumor SUV_mean_ values is small and clinically rather non-relevant.

The Biograph Vision Quadra PET/CT system essentially is comprised of an axial concatenation of the equivalent of four Biograph Vision PET 600 scanners, providing an axial FOV of 106 cm, equipped with silicon-based photomultiplier (SiPM)-based photon detectors characterised by superior timing resolution. These characteristics enable improved TOF estimation and efficient photon detection, a high spatial resolution, and an increased sensitivity by a factor of five when compared to the preceding Biograph Vision 600 system [25–28]. The results of the visual analysis of our study show that this increased sensitivity of the new scanner, may allow for a reasonable decrease in acquisition time by 5 min (i.e., from 10- to 5-min), even when applying low dose examination protocols. Further, absolutely no differences were observed between the reference of 10-min PET protocol and the 8-min and 6-min acquisitions regarding classification of scans as normal or pathological, while the differences in lesion detectability rate were minimal and, importantly, without any potential clinical consequence for any patient of the cohort. This means that all patients would have been correctly characterized in terms of staging and restaging of the disease even when applying a 6-min, low-dose PET/CT protocol. Notably, with regard to the proportion of pathological scans, a decrease of the acquisition times even up to four minutes did not lead to any significant change, as reflected by the respective 95%-CI for the differences between various acquisition prortocols.

Particularly in malignant melanoma, where true whole-body (total body) imaging and, therefore, two-bed position scanning with the Biograph Vision Quadra system is required, this shortening of PET acquisition times without a concomitant, clinically relevant decrease of diagnostic performance would be very practical for busy departments, since it would allow the execution of a greater number of exams and lead to an increase in patient throughput. At the same time, the shortened protocol would improve patient comfort and considerably prevent motion artifacts. Moreover, the reduction of the applied radiopharmaceutical dose represents an economic as well as a dosimetric advantage. These considerations take on even greater significance, when taking into account the potential need for serial PET/CT scanning of many of the patients with advanced melanoma who undergo immunotherapy in terms of treatment monitoring [29].

On the other side, the shortening of the PET protocol was associated with a decrease of both parameters used to objectively evaluate image quality, namely SNR and TBR. The observation that extending the acquisition times improves image quality is not new. In specific, the herein observed trend is in line with the results of previous studies also employing ultra-extended FOV PET/CT scanners, which similarly showed a decreasing SNR as PET acquisition times decreased in cohorts of different tumors studied with different tracers applied both at standardized and low doses [13–15].

The parameter TBR also decreased progressively from PET-10 to PET-2. This finding seems to be inconsistent with previous studies on ultra-extended FOV PET/CT scanners, which showed an inverse relationship, i.e., higher TBR values in shorter acquisitions compared to standard acquisitions [13, 15]. It has to be noted, however, that in these studies, TBR was calculated as the ratio of SUV_max_ of tumor lesions divided by SUV_mean_ of the liver background, which was not the case in our analysis. In the present analysis TBR was defined as the ratio of SUV_mean_ of the melanoma lesions divided by SUV_mean_ of the liver. We prefered to calculate the mean tumor uptake since it incorporates information from multiple voxels, making it less susceptible to image noise compared to SUV_max_ [30]. This approach of SUV_mean_-based calculation of TBR was also recently applied by Sari et al. in another study with the Biograph Vision Quadra system [31]. Indicatively, the application of the alternative formula (lesion SUV_max_/liver SUV_mean_) would have led to consistent results with the above-mentioned studies, namely a progressive increase of TBR as the acquisition times decrease (data not provided in the manuscript but available at request). Notably, in lesion-based analysis, the reduction of acquisition time to 6 min and 5 min led to no significant differences in TBR and tumor lesions’ SUV_mean_, respectively.

Our study has some limitations. Firstly, this is a single-center retrospective analysis. Thus, a validation of the herein presented findings in larger patient cohorts ideally studied in the context of a multicenter, prospective trial would be required. Secondly, the use of histopathological findings as a reference to validate the detectability of tumor lesions is a more reliable method than referring to PET-10 images. However, in our study the vast majority of the PET-positive findings were not histopathologically confirmed, which is, obviously, not possible in the clinical setting and was, moreover, beyond the scope of this analysis. Nevertheless, since all included patients had a biopsy-confirmed melanoma, for which they received the respective treatment, and given the very high accuracy of [^18^F]FDG PET/CT in detection of the malignancy, most focal [^18^F]FDG-avid lesions, for which a benign aetiology could be safely excluded, were considered consistent with tumor involvement. Thirdly, the analysis was confined to the comparison of the 10-min PET scans, as recommended by the manufacturer, with five specific sub-divisions (2-min, 4-min, 5-min, 6-min, and 8-min) of the original acqusitions, and did not include even more acquisitions. However, we believe that the conclusions drawn with the present comparisons provide sufficient hints for clinical practice in the context of the purpose of this study. Finally, the study focused on the analysis of the scans covering the first bed position, i.e. from the head to the thigh. The PET scans performed in terms of the second bed position, covering the lower extremities, were not analyzed in the same fashion, since the original PET acquisitions lasted shorter (5 min). This will be however the topic of a future work of our group.

## Conclusion

In an attempt to determine an appropriate acquisition time range for low-dose oncological imaging with the new LAFOV Biograph Vision Quadra PET/CT, we investigated a cohort of malignant melanoma patients after administration of body weight-adapted dose of 2.0 MBq/kg [^18^F]FDG. We could show that the reduction of PET acquisition times from head to upper thigh up to 5 min in low-dose LAFOV PET/CT imaging can be safely performed, leading to no significant changes in terms of visual analysis. Moreover, the shortening of the PET protocol from 10 to 6 min was associated with absolutely no potential clinical consequences for any of the enrolled patients. Finally, an increase in noise was observed by reducing the protocol from longer to shorter acquisition times, although in lesion-based analysis, the change in TBR and tumor SUV_mean_ values was non-significant up to 6-min and 5-min acquisitions, respectively. These findings may have significant implications for the diagnostic work-up of patients with melanoma, given the need for true whole-body imaging in this type of cancer.

## Supplementary Information

Below is the link to the electronic supplementary material.

Supplementary file1 (DOCX 16 KB)

## Data Availability

The datasets generated during and/or analyzed during the current study are available from the corresponding author on reasonable request.
