# Supplementary material for: Application of the long axial field-of-view PET/CT with low-dose [18F]FDG in melanoma
Source: Eur J Nucl Med Mol Imaging. 2022 Dec 7;50(4):1158–67. doi: 10.1007/s00259-022-06070-7 (PMC9931831; doi:10.1007/s00259-022-06070-7)
Supplement: Supplementary file 1 — Supplementary file1 (DOCX 16 KB) [file 259_2022_6070_MOESM1_ESM.docx]

**Supplementary Table 1** Quantitative parameters of the background and tumor lesions in terms of the objective evaluation of PET/CT image quality. Data are presented as mean ± standard deviation (mean ± SD).

| **Measurement** | **PET-10** | **PET-8** | **PET-6** | **PET-5** | **PET-4** | **PET-2** |
| --- | --- | --- | --- | --- | --- | --- |
| Liver SUV_mean_ | 2.61 ± 0.61 | 2.61 ± 0.62* | 2.62 ± 0.62* | 2.63 ± 0.62* | 2.63 ± 0.62* | 2.64 ± 0.61* |
| Liver SUV_max_ | 3.31 ± 0.77 | 3.38 ± 0.81* | 3.52 ± 0.83* | 3.61 ± 0.87* | 3.73 ± 0.95* | 4.29 ± 1.07* |
| Liver SD | 0.25 ± 0.08 | 0.27 ± 0.08* | 0.31 ± 0.09* | 0.34 ± 0.10* | 0.38 ± 0.12* | 0.52 ± 0.15* |
| Liver SNR | 10.83 ± 2.11 | 9.82 ± 1.79* | 8.60 ± 1.60* | 7.94 ± 1.44* | 7.18 ± 1.29* | 5.25 ± 1.13* |
| Mediastinal SUV_mean_ | 2.05 ± 0.53 | 2.07 ± 0.55* | 2.09 ± 0.55* | 2.10 ± 0.55* | 2.10 ± 0.55* | 2.12 ± 0.53* |
| Mediastinal SUV_max_ | 2.54 ± 0.72 | 2.61 ± 0.76* | 2.71 ± 0.79* | 2.79 ± 0.76* | 2.85 ± 0.75* | 3.22 ± 0.85* |
| Mediastinal SD | 0.23 ± 0.09 | 0.25 ± 0.10* | 0.29 ± 0.10* | 0.31 ± 0.11* | 0.34 ± 0.12* | 0.47 ± 0.16* |
| Lesion-based tumor SUV_mean_ | 7.13 ± 4.61 | 7.14 ± 4.57 | 7.11 ± 4.53 | 7.07 ± 4.49 | 7.02 ± 4.47* | 6.90 ± 4.40* |
| Lesion-based tumor SUV_max_ | 10.95 ± 8.27 | 11.05 ± 8.32* | 11.17 ± 8.44* | 11.23 ± 8.46* | 11.27 ± 8.50* | 11.69 ± 8.72* |
| Lesion-based TBR | 2.88 ± 1.98 | 2.88 ± 1.94 | 2.85 ± 1.90 | 2.83 ± 1.87* | 2.81 ± 1.86* | 2.75 ± 1.82* |
| Patient-based tumor SUV_mean_ | 6.72 ± 4.32 | 6.75 ± 4.34 | 6.71 ± 4.35 | 6.69 ± 4.31 | 6.63 ± 4.27* | 6.51 ± 4.12* |
| Patient-based tumor SUV_max_ | 10.25 ± 7.53 | 10.36 ± 7.69* | 10.46 ± 7.85* | 10.52 ± 7.87* | 10.54 ± 7.83* | 10.92 ± 7.93* |
| Patient-based TBR | 2.74 ± 2.11 | 2.74 ± 2.10 | 2.71 ± 2.09* | 2.70 ± 2.09* | 2.67 ± 2.07* | 2.62 ± 1.98* |

*SUV_mean_*, mean standardized uptake value; *SUV_max_*, maximum standardized uptake value; *SD*, standard deviation; *SNR,* signal-to-noise ratio; *TBR,* tumor-to-background ratio.

SNR of the background was measured as liver SUV_mean_ divided by liver SD

TBR was defined as tumor lesion SUV_mean_ divided by liver SUV_mean_

*Significant difference in comparison with the respective values of PET-10, p < 0.05
